# Supplementary figures and images for: High-risk human papillomavirus prevalence among South African women diagnosed with other STIs and BV
Source: PLoS One. 2023 Nov 30;18(11):e0294698. doi: 10.1371/journal.pone.0294698 (PMC10688634; doi:10.1371/journal.pone.0294698)

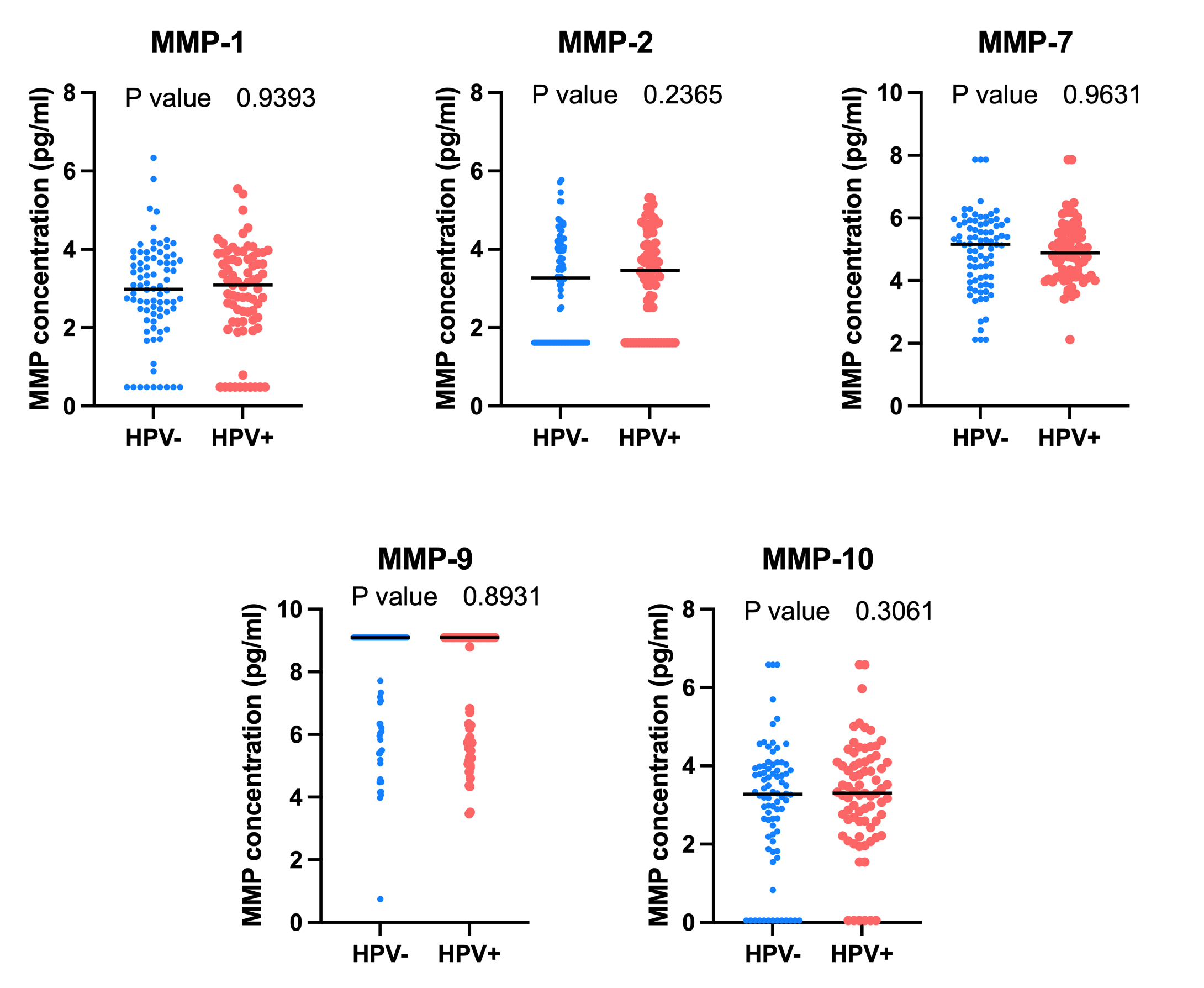

Supplement: S1 Fig — The t-test was used to compare median log-transformed MMP concentrations between HPV-infected and uninfected groups. (TIF) [file pone.0294698.s001.tif]
